# A cross sectional study evaluating screening using maternal anthropometric measurements for outcomes of childbirth in Ugandan mothers at term

**DOI:** 10.1186/s13104-015-1183-z

**Published:** 2015-06-02

**Authors:** Ian G. Munabi, Samuel Abilemech Luboga, Florence Mirembe

**Affiliations:** Department of Human Anatomy, School of Biomedical Sciences, Makerere University College of Health Sciences, New Mulago Hospital Complex, P. O. Box 7072, Kampala, Uganda; Department of Obstetrics and Gynaecology, School of Medicine, Makerere University College of Health Sciences, New Mulago Hospital Complex, Kampala, Uganda

**Keywords:** Childbirth, Anthropometry, Pelvimetry, Height, Weight, Pelvis height, ROC analysis

## Abstract

**Background:**

Birth related newborn and maternal mortality/morbidity remains high in most of sub-Saharan Africa compared to the rest of the world. In this low income region there is a need for valid, low cost, easy to use mass screening tests. This study looked at the screening value of maternal: height, weight and pelvis height, for assessing the outcomes of parturition in Ugandan mothers at term.

**Methods:**

This was a multi site cross-sectional study on mothers with singleton pregnancies in labour at various hospitals in different parts of Uganda. A summary of the details of the pregnancy, maternal height, weight and the delivery record were captured and analysed to generate descriptive and inferential (multilevel logistic regression analysis) and diagnostic (Receiver Operator Curve analysis) statistics.

**Results:**

We recruited 1146 mothers from all the study sites during the study period of whom 987 (86.13%) had normal deliveries and healthy babies. Mothers with adverse outcomes included 107 mothers that had caesarean section and 52 mothers who had vaginal deliveries with foetal Apgar score of ≤7 at 5 min of whom 11 had fresh still births. Maternal height (Adj OR 0.97, 95% CI 0.94–1.00) and maternal pelvis height (Adj OR 0.73, 95% CI 0.61–0.86) were significantly associated with adverse pregnancy outcomes. The combination of maternal: height (<150 cm), weight (>55.7 kg) and pelvis height (>8.95 cm) had the best diagnostic value with a combined area under the curve of 0.60.

**Conclusions:**

It was observed that an increase in either maternal pelvis height or maternal height was associated with a significant reduction in adverse pregnancy outcomes. The cut off values of all three evaluated maternal anthropometric measurements were of low test accuracy as screening tests even when used together. Further research is needed to develop low cost screening tools for use in low income settings.

## Background

Death of new born infants remains a major problem for most of the Low and Middle Income Countries (LMICs) where these newborn deaths account for a large proportion of the under 5 child mortality [1]. According to Lawn et al. [2], intrapartum related neonatal deaths account for 10% of deaths in children under 5 years of age globally. Also globally it is estimated that macerated still births account for an estimated 17% of all still births, meaning that most (83%) of the estimated 3.2 million annual still birth deaths occur during the intrapartum period as fresh still births [2, 3]. In Sub-Saharan Africa estimates show that 27% of all the under five deaths are due to still births [4]. These high proportions of child deaths are associated with equally high rates of maternal morbidity and mortality. Hogan et al., observed that three sub-Saharan African countries: Nigeria, Democratic Republic of Congo, and Ethiopia, were among the six countries accounting for more than 50% of the global maternal deaths [5]. In local study at the national referral hospital in Uganda an early neonatal death rate of 109 deaths per 1,000 live births was observed, mainly attributed to respiratory distress and poor foetal monitoring during labour [6]. These high maternal and child mortalities are also indicative of the delays and low quality of care received by mothers prior to and during parturition [7]. This means that interventions that will improve the quality of birth care can contribute to a reduction in: early neonatal death, maternal morbidity and mortality [8, 9]. This is what has driven the development of technologies like ultrasound and more recently a renewed interest in doing pelvimetry as a method to improve prediction of the parturition outcomes [10, 11].

**Figure 1:**
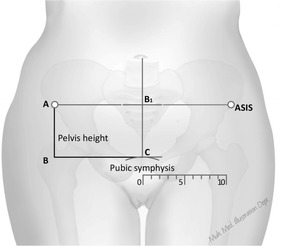
Pelvis height using surface landmarks on the human body.

In low income settings the absence of reliable electricity supply coupled with the fact that most of the population is poor, rural and more likely to be attended to by low cadre of health workers or unskilled birth attendants [12], makes a low cost, easy to use test, a preferred option for mass screening of pregnant mothers. It is such concerns that formed the basis for World Health Organizations’: Affordable, Sensitive, Specific, User friendly (simple to use), Rapid, Equipment free, and Delivered to those that need it most (ASSURED) characteristics of an ideal diagnostic test for resource limited settings [13]. In this study we focused our exploration on three easy to obtain anthropometric measurements. These included maternal height and weight that are already being routinely collected and a new measure of maternal pelvis height. Pelvis height, is already being used by motor vehicle engineers to delineate the contribution of the pelvis to the total height of the individual [14]. Pelvis height was obtained using two easy to locate bony surface land marks the anterior superior iliac spines and top of the symphysis pubis (see Figure 1). This study set out to assess the screening value of maternal: height, weight and pelvis height on the outcomes of parturition in a cohort of Ugandan mothers at term.

## Methods

This was a multi site cross-sectional study carried out at purposively selected health facilities from different geographical regions and levels of Ugandas health care system. We made this selection of hospitals to cater for geographical distribution of known major ethinic groupings within Uganda [15, 16]. First we included Mulago hospital the National Tertiary Care and Referal Teaching hospital in cental Uganda. Second Arua regional referal hospital in North western Uganda and Anaka (North cental Uganda). Third we included private not for profit level IV health center hospitals, Kumi (Eastern Uganda), St. Josephs Kitgum (extreme North central Uganda), Kilembe (Western Uganda), and Nyakibale (Southwestern Uganda). Each of these hospitals was capable of handling cesarean delivery with a minimum of 150 mothers delivered per month. We included Mothers in active labour, having pregnancies of ≥37 weeks estimated using the symphysio-fundal height, came to the above listed sites during the 14 months of the study starting January 2013, gave an informed consent, having a live singleton baby with no obvious congenital abnormalities after childbirth, and baby with cephalic presentation before childbirth. We excluded mothers with history of previous cesarean section and those with medical conditions like hypertension or diabetes or carrying a multiple pregnancy.

The mothers were recruited consecutively on a daily basis from each of the centers by a team of previously trained midwifes. The target sample size of 933 mothers was calculated to give a minimum AUC of 0.555, obtained from our pilot data, compared to the NULL (0.500) for equivalent sized sub-groups, a β = 0.9 and α = 0.05 using the calculator for sample size calculation based on Area under the curve available online at (https://www.statstodo.com/SSiz2ROCs_Pgm.php). This was inflated by a design effect of 1.2 for the 7 sites to give a final total sample size of 1,120 participants [17].

For each mother we collected data on age in years, height in centimeters and weight in kilograms measured using the available hospital equipment [18], gravidity (current pregnancy + all previous pregnancies), fetal presentation, head descent in terms of number of 5ths engaged and symphysio-fundal height in centimeters, a proxy for gestational age on clinical examination to the nearest 0.1 cm. After delivery we also collected data on the mode of delivery, sex of the baby, APGAR scores, presence of caput, meconium staining, whether augmentation or stimulation of labour was done, birth weight in kilograms and whether the mother had a cesarean section at the facility. For each mother the pelvis height in centimeters was measured twice, at the time of admission by the attendant midwife, using the anterior superior illiac spine (ASIS) and the Symphysis pubis bony body landmarks using a pair of transparent rigid rulers placed at right angles to each other, as demonstrated in Figure 1 (see lines AB and BC). The average of these two measurements was used for analysis. We trained the midwifes at each site on how to measure pelvis height and complete the study questionnaire at the start of the study. There were additional refresher training sessions during the site visits by IGM. The training, which in addition placed emphasis on proper fetal monitoring, partogram use and obtaining informed consent, was accompanied with supervised hands on practice for each midwife until they demonstrated competency to take the measurements. At each site the incharge was recruited as the site supervisor for the duration of the study to ensure that all forms were completed, measurements done properly and to provide training support for midwifes that were on duty after the 1 day training sessions.

Data was entered into Epidata version 3.2 (Epidata association, Denmark) and exported to STATA 12 (StataCorp LP, Texas, USA) for eventual analysis. Paired correlation coefficients were determined for each of the study variables highlighting significant correlations. Logistic regression was used to identify predictors of unfavourable outcomes of labour that included: delivery by cesarean section, vaginal delivery of a baby with an APGAR score of ≤7 at 5 min [19] or mother being referred. To obtain predicted probabilities, all the variables with the optimal cut-off that were significant at the 0.20 level in the univariate analysis were included in the subsequent modeling [20]. To cater for the study design and variability in recruitment numbers from the different sites multilevel logistic regression using the gllamm function in STATA was used to calculate the odds ratios with iterations nipped at 60 for final modeling [21]. The sensitivity and specificity of each anthropometric measurement was computed, curves plotted, and optimal cut-off values with out cost considerations identified for each variable using Receiver Operator Characteristic (ROC) using MedCalc Statistical Software version 13.0.6 (MedCalc Software bvba, Ostend, Belgium; http://www.medcalc.org; 2014). All simulations for the ROC analysis were done up to 1000 times using the bootstrapping procedures. During analysis, cut off values were generated as part of the ROC analysis with added comparisons with values from literature for maternal weight, maternal pelvis height [22] and maternal height [20, 23]. No distinction was made between normal vaginal and instrumental vaginal deliveries that were grouped together, during analysis, as a single outcome measure, vaginal delivery. The group with favourable outcome was defined as a vaginal delivery with a healthy live infant. Any deviation from having a vaginal delivery resulting into a healthy live infant was defined as an unfavourable outcome. Mothers’ with multiple outcomes were counted only once and categorised as having had an unfavourable outcome in the event that this was observed. The *T* test was used to obtain the level of significance for differences in the means of the study variables for the normal vaginal delivery with normal baby group and unfavourable outcomes group. Any observation found with a missing value was dropped from analysis. A P < 0.05 was considered significant for all tests.

Ethical approval was obtained from Makerere University School of Biomedical Sciences IRB and Uganda National of Science and Technology. At each site we verbally obtained the consent of the participating nursing staff to be part of the study and offered an equivalent of one United States dollars compensation for each birth record filled to completion. The participating mothers, each gave informed consent after explanation of the study and signed the informed consent form to participate in the study on admission. Informed consent was obtained by the attending midwife from mothers. In Uganda, the Uganda National Council of Science and Technology (UNCST) allows emancipated minors to consent to participate in research as long as they have been informed about the risks involved [24]. Authors considered minors as emancipated adults and all mothers were free to consult their spouses or next of kins since the study required one to provide contact information as part of the consent process. With the exception of measuring maternal pelvis height there were no other procedures or modifications made to the current birthing practice at any of the participating sites. Refusal to participate in the study did not result in a mother being denied access to health care or required services at the participating facility. No identifier marks of personal information was used in the analysis and subsequent reporting of the study results.

## Results

We recruited 1,146 mothers from all the study sites during the study period of whom 987 (86.13%) had normal deliveries and healthy babies. The adverse pregnancy outcomes were categorized as follows: 107 had caesarean section births, 52 vaginal deliveries with an Apgar score ≤7 of who 11 had fresh still births. Table 1 provides a summary of birth outcomes at each site contains totals of some observations that are higher than the total study population due to some mothers having more than one outcome. In Table 2, significant differences were observed in the comparisons between the two groups of mothers on the following variables: maternal weight, gravida (number of pregnancies), meconium staining, and presence of caput, APGAR score, birth weight and body mass index (BMI) for these two groups of outcomes. Significant correlations were observed with: maternal age and maternal height (0.20, P < 0.001), maternal age and maternal weight (0.24, P < 0.001), maternal age and gravida (0.74, P < 0.001), maternal weight and maternal height (0.36, P < 0.001), gravida and maternal height (0.17, P < 0.001), gravida and maternal weight (0.15, P < 0.001), symphysio-fundal height and maternal height (−0.12, P < 0.001), foetal head descent and maternal height (−0.12, P < 0.001), symphysio-fundal height and head descent (0.16, P < 0.001), caput presence and symphysio-fundal height (0.13, P < 0.001), meconium staining and symphysio-fundal height (0.18, P < 0.001), meconium staining and maternal height (−0.23, P < 0.001), meconium staining and maternal weight (0.16, P < 0.001), meconium staining and APGAR score at 5 min (−0.14, P < 0.001), meconium staining and presence of capture (0.39, P < 0.001), meconium staining and augmentation (0.18, P < 0.001), augmentation and presence of caput (0.38, P < 0.001), birth weight and maternal age (0.23, P < 0.001), birth weight and maternal weight (0.19, P < 0.001), birth weight and gravida (0.24, P < 0.001), birth weight and meconium staining (0.16, P < 0.001). Significant correlations were observed for the study variable, pelvis height and: caput presence (0.18, P < 0.001), meconium staining (0.17, P < 0.001), gravida (0.21, P < 0.001), and symphysio-fundal height (0.12, P-value 0.004). Only the correlation between maternal age and gravida (current pregnancies + past deliveries) (0.74, P < 0.001) was significantly large.

**Table 1:** Summary of births from each participating study site

Hospital	1	2	3	4	5	6	7	Total
Vaginal delivery	282	28	79	52	145	323	116	1,025^a^
Caesarean births	20	5	9	5	17	36	15	107
Fresh still birth	1	0	0	0	1	9	0	11
APGAR ≤7	3	0	6	6	6	28	3	52
Total	306	33	94	63	169	396	134	1,195^a^

Key for hospitals: 1 Old Mulago, 2 Kilembe, 3 Nyakibale, 4 Anaka, 5 Kitgum, 6 Arua, 7 Kumi.

^a^Note some mothers had more than one outcome thus being counted more than once in this table.

**Table 2:** Comparison of participant’s observations with respect to pregnancy outcomes

Variable	No.	Combined	Normal delivery		Unfavourable outcome		
		Mean (SD)	No	Mean (SD)	No	Mean (SD)	T test (P-value)
Maternal age	1115	25.36 (5.96)	958	25.33 (5.74)	157	25.55 (7.17)	−0.43 (0.67)
Maternal height	1092	158.90 (9.53)	942	159.09 (9.53)	150	157.74 (9.48)	1.61 (0.11)
Maternal weight	1106	63.75 (9.78)	952	64.14 (9.76)	154	61.28 (9.52)	*3.38* (*0.01*)
Maternal pelvis height	1117	7.32 (1.89)	959	7.28 (1.85)	158	7.58 (2.09)	−1.84 (0.07)
Gravida	1081	3.07 (2.15)	931	3.01 (1.99)	150	3.45 (2.95)	*−2.34* (*0.02*)
Symphysio-fundal height	1121	38.19 (0.72)	968	38.19 (0.71)	153	38.16 (0.80)	0.45 (0.65)
Foetal head descent	1022	0.97 (0.18)	878	0.97 (0.16)	144	0.94 (0.23)	1.70 (0.09)
Augmentation	1033	0.07 (0.25)	914	0,07 (0.25)	119	0.08 (0.27)	−0.41 (0.68)
Sex of baby	1016	0.53 (0.50)	893	0.52 (0.50)	123	0.57 (0.50)	−0.96 (0.34)
Meconium stained liquor	1083	0.47 (0.64)	948	0.41 (0.58)	135	0.91 (0.82)	*−8.88* (<*0.01*)
Caput present	1062	0.14 (0.35)	933	0.12 (0.32)	129	0.33 (0.47)	*−6.71* (<*0.01*)
APGAR Score at 5 min	1085	9.41 (3.31)	954	9.54 (3.36)	131	8.44 (2.71)	*3.57* (<*0.01*)
Birth weight	1105	3.18 (0.52)	973	3.17 (0.50)	132	3.31 (0.59)	*−2.93* (<*0.01*)
BMI	1087	25.34 (4.14)	937	25.44 (4.23)	150	24.69 (3.44)	*2.06* (*0.04*)

Significant values are in italics (P < 0.05).

Table 3 provides a summary of the logistic regression modelling in which the final model, controlling for the hospital from which each participant was recruited, had a significant 3% reduction in odds of adverse maternal outcomes for each centimetre increase in maternal height (Adj OR 0.97 95% CI 0.94–1.00). There was also a 27% significant reduction in the odds of having a poor outcome for each centimetre increase in maternal pelvis height (Adj OR 0.73 95% CI 0.61–0.86). Maternal weight (Adj. 0.98 95% CI 0.95–1.01) and the number pregnancies (Adj OR 1.09 95% CI 0.99–1.20) though retained in the model were not significant. For the foetus: there was a threefold increase in adverse birth outcomes with the presence of a meconium stained liquor (Adj. OR 2.45 95% CI 1.66–3.61), about 30% reduction in odds for adverse outcomes for each unit increase in APGAR score at 5 min (Adj. OR 0.68 95% CI 0.57–0.86), and a threefold increase in the odd of an adverse outcome for each kilogram increase in birth weight (Adj. OR 3.10 95% CI 1.91–5.01).

**Table 3:** Multilevel Logistic regression analysis of the various variables and the outcome

Variable	OR	95% CI	P-value	Adj. OR	95% CI	P-value
Maternal Age	1.00	0.98–1.03	0.75	–	–	–
Maternal Height	0.98	0.96–1.00	*0.04*	0.97	0.94–1.00	*0.04*
Maternal Weight	0.98	0.96–1.00	*0.02*	0.98	0.95–1.01	0.13
Maternal Pelvis height	0.85	0.75–0.96	*0.01*	0.73	0.61–0.86	<*0.01*
Gravida	1.04	0.96–1.12	0.28	1.09	0.99–1.20	0.09
Symphysio-fundal height	0.91	0.71–1.16	0.44	–	–	–
Foetal head descent	0.42	0.17–1.04	0.06	–	–	–
Augmentation	0.86	0.40–1.84	0.71	–	–	–
Sex of baby	1.27	0.85–1.90	0.24	–	–	–
Meconium stained	3.79	2.69–5.34	<*0.01*	2.45	1.66–3.61	<*0.01*
Caput present	2.93	1.87–4.61	<*0.01*	–	–	–
APGAR Score at 5 min.	0.64	0.56–0.74	<*0.01*	0.68	0.57–0.86	<*0.01*
Hospital	1.13	1.04–1.25	<*0.01*	–	–	–
Birth weight	2.16	1.49–3.13	<*0.01*	3.10	1.91–5.01	<*0.01*

Significant values are in italics (P < 0.05).

**Figure 2:**
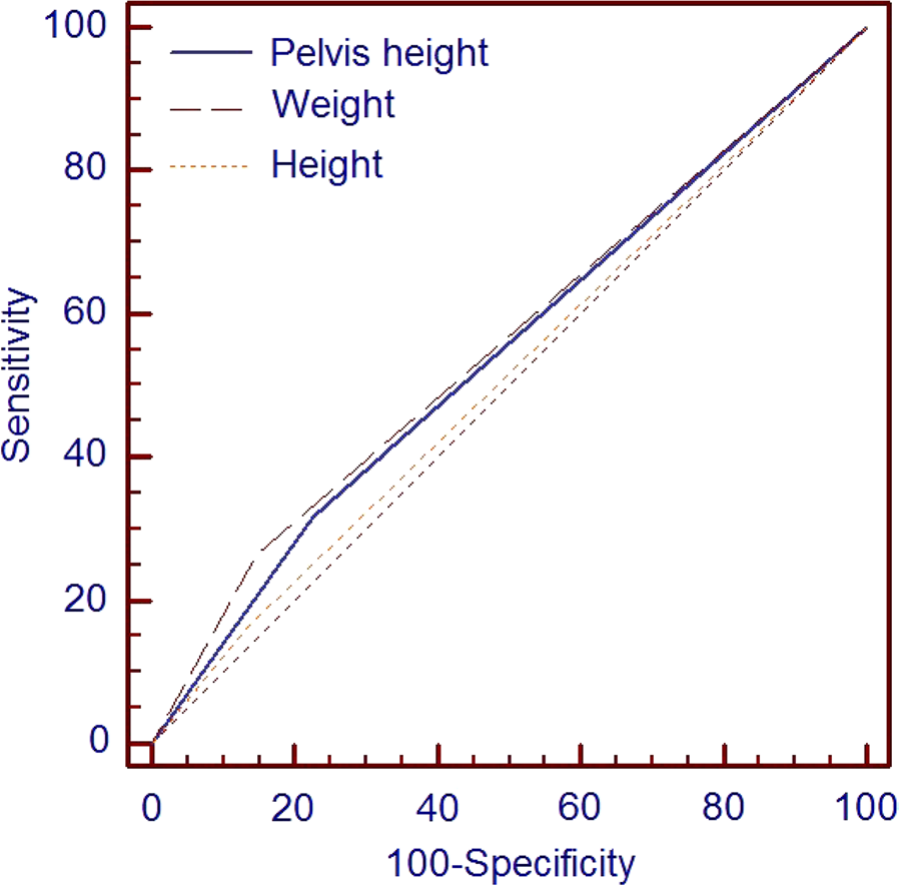
ROC plots for maternal height, weight and pelvis height cut offs.

Table 4 summarises the ROC curve analysis of the three anthropometric measurements: maternal height, maternal pelvis height and maternal weight. In this Table 4, it is important to note that there was no significant difference in the Area under the Curve (AUC) between each of these variables as confirmed in Figure 2. A test combination of maternal height (<150 cm) and pelvis height (8.95 cm) performs better than maternal height (<150 cm) alone (combined: LR+ 1.7 LR− 0.86, AUC: 0.55, SE: 0.02, 95% CI 0.53–0.58). The combination of the three anthropometric measurements using the cut offs of: maternal height (<150 cm), maternal weight (>55.7 kg) and maternal pelvis height (8.95 cm) performs even better than maternal height (<150 cm) alone (combined: LR+ 2.99 LR− 0.75, AUC: 0.60, SE: 0.02, 95% CI 0.57–0.63). Included in Table 4 note the additional cut off of 7.50 cm for pelvis height obtained from previous literature [22]. There was no significant difference in the AUC between the cut off of 7.5 cm from literature and the one obtained from this study of 8.95 cm (P = 0.72). Also in Table 4, note the significant difference in AUCs for cut off points of maternal height (<150 cm) and maternal height (<160.4) (P < 0.01).

**Table 4:** Showing ROC performance values for optimal thresholds of pelvis height, weight and Height

Threshold (cm)	Sensitivity	Specificity	LR+	LR−	AUC (SE, P-value^a^)
Pelvis height >8.95	30.68	77.64	1.37	0.89	0.54 (0.02, 0.02)
Pelvis height >7.5^b^	49.44	55.63	1.11	0.91	0.53 (0.02, 0.21)
Height <150^c^	15.73	87.27	1.24	0.97	0.52 (0.01, 0.30)
Height <160.4	69.64	43.06	1.22	0.70	0.56 (0.01, <0.01)
Weight (kg) >55.7	26.16	85.10	1.76	0.87	0.56 (0.02, <0.01)

^a^P-value for comparison to the 0.5 ROC line.

^b^Recomended cut off threshold from literature.

^c^Current practice threshold cut off for maternal height.

## Discussion

We set out to evaluate the value of screening mothers for pregnancy adverse outcomes at parturition using maternal height, weight and pelvis height in a cohort of Ugandan mothers at term. We found that increases in either maternal pelvis height or maternal height were associated with a significant reduction in odds for an adverse outcome of pregnancy keeping all the other factors constant. Maternal pelvis height had previously hypothesized to have an effect on the outcomes of labour in African populations by Zivanovic and Leister [22, 25]. Our study, which had a larger sample size than the previous two studies, confirms that increasing maternal pelvis height is associated with reduced risk of adverse pregnancy outcomes. The performance of pelvis height as a screening tool was further confirmed by the more appropriate ROC analysis [26]. According to Swet, an AUC of 1.0 denotes perfect test discrimination on ROC analysis. AUC values of 0.5–0.7 indicate low test accuracy, 0.7–0.9 indicate moderate test accuracy and 0.9–1.0 high test accuracy [27]. Our study showed that maternal pelvis height, which is obtained using two easy to identify bony surface landmarks [the symphysis pubis and the anterior superior iliac spines, (see Figure 1)], has a low test accuracy for screening mothers for adverse outcomes of pregnancy in low income settings.

Bisig et al., suggested that screening tests perform better if used together to make decisions on the management of patients [28]. This means that a decision on the management of a mother is based on the results of all three tests applied at the same time also referred to as using the tests in parallel. We have shown that indeed the additive power of these tests is best achieved with the combination of maternal: height and weight and pelvis height used together simultaneously as a package for the assessment in a single examination [28]. The implication of this approach is that a negative result on any one of the three tests used in combination would lead to remedial action on the part of the health care worker.

Maternal weight is influenced by several factors that include cultural modification of maternal feeding habits during pregnancy and the mothers’ social economic status. This greater sensitivity to changes in feeding during pregnancy on top of the effects of pregnancy are some of the reasons maternal weight is listed among the measures for antenatal screening by the WHO [29–32]. The other concern with maternal weight and feeding is their association with birth weight especially in the last months of pregnancy. Rapid weight gain in this period is also associated with among others: greater birth weight, which in this study was associated with a threefold increase in the odds for an adverse outcome for each additional kilogram in any two mothers with the same anthropometric measurements value for all the other variables in the model. This may explain the persistent non significance of maternal weight observed in this study.

Maternal height was observed to have a significant association with adverse outcomes of pregnancy while maternal weight was not significant on the logistic regression modelling. It is important to note that the significance of maternal height was less than that of maternal pelvis height. This could be explained by the observation that in females the long bone growing points will fuse between the age of 16 and 18 years of age while the pubic bone of the human female pelvis keeps growing till the age of 28–30 years leading to increased vertical and horizontal diameters of the birth canal [33]. This observation becomes more significant given that the majority of the mothers in this study were still in their 2nd decade of life thus having changing pelvis dimensions compared with their already fixed maternal heights. According to literature, short maternal height is an indicator of long standing malnutrition, and more relevant to the Asian than African continent. The African continent has more of early childhood protein energy malnutrition as a result of dietary imbalance [34]. Despite being a good indicator of chronic malnutrition [34], maternal height is also not listed among the recommended indicators for use in antenatal screening by the World Health Organisation due to its low predictive power [29–32]. In low income settings the continued use of maternal height is supported by: the continued persistence of childhood malnutrition and infections that limit or divert the supply of nutrients leading to permanent loss in bone growth which requires more than one generation to correct [35], and the absence of alternative low cost and accurate tools for screening of mothers. Ignoring the logistic regression analysis as advised by Pepe et al. [26], maternal height with the current cut off of 150 cm has a low test accuracy AUC [27] (see Table 4). Thus given the ethical challenges of not screening in a population with high maternal and neonatal mortality rates as described earlier, the continued use of maternal height alone or in combination with other anthropometric measurements is justified in these low income settings.

The clinical utility of maternal height can be improved by addition of other diagnostic tests as has been demonstrated above or by adjustment of the related cut off values for screening. Studies done in other settings have recommended the use of maternal height cut offs of <155.5 cm [20], and <162.5 cm [23] for African mothers. Table 4 shows that increasing the maternal height cut off to the ROC optimum cut off of <160.4 cm improves the sensitivity of the test while increasing the false positive rate. The observed low values for the different AUCs for the different tests (see Table 4), further demonstrates the relevance of using a combination of tests based on maternal: height, weight and pelvis height to increase the diagnostic value of the assessment. As has been noted above the combination of the three measurements maternal height, maternal pelvis height and maternal weight, has a greater AUC of 0.6. Despite this being a low test accuracy value according to Swet [27], it remains important given the low utilization of the health care facilities in our low income settings [12] and the ethical challenges highlighted above. Also given the very high value placed on having a vaginal delivery [36], there remains a critical need for diagnostic tools to guide screening and eventual decision making process on where mothers go for childbirth. This means that decisions based on assessments from a combination of tools that utilize easy to use anthropometric measurements as proposed in this paper may make the difference between life and death for either the mother and or her unborn child. This is what makes the low cadre health worker in these low resource settings facilities, where most of the maternal and child mortality/morbidity are observed, the ideal target users for this combination of screening measurement tools.

The limitations of this study include the use of anthropometric measurements obtained from real world settings using basic tools with the inherent potential for human error despite the training. The non-random sample used may have introduced selection bias in the study, which may have led to over- or under-reporting of the accuracy of the various measurements/outcomes thus requiring the cautious extrapolation of the study findings to the general population even with the fairly large sample size and the random nature of onset of labour. It should also be noted that background variation in diet which influences individual mothers growth patterns [37] and prior teenage pregnancies [38] may have resulted in a less uniform study population as evidenced by the observed significant correlations between maternal age, maternal height and gravidity. Finally in these settings the majority of mothers prefer to deliver at home as opposed to in the health facilities [12] further limiting the representativeness of the sample in relation to the population in the community. In this study training of the health workers performing the measurements in addition to the requirement to do the measurement of pelvis height twice with a transparent ruler were used to reduce human error. Simulation using the bootstrapping routine was used to make the confidence intervals more representative. In spite of these limitations the study findings provide useful information from a real world working environment on the effectiveness of the three anthropometric measurements as screening tools. The information generated in this study is relevant for the screening of mothers in remote rural settings where expertise is limited and the need for such tools to guide early referral decisions is most in demand.

## Conclusions

This study identified maternal pelvis height as an additional significant anthropometric measurement for use in screening mother for adverse outcome of pregnancy. The three maternal anthropometric measurements cut offs include: height (<150 cm), weight (>55.7 kg) and pelvis height (>8.95 cm) all had low accuracy as screening tests on ROC analysis despite having strong significant associations with the adverse outcomes of pregnancy. Their predictive value is increased when used simultaneously although this is still of low test value accuracy. Additional studies are required to further assess these tools to support actual screening of mothers in low income settings.

## Abbreviations

AUC: Area under the curve
ICC: Intra class correlation coefficient
IRB: Institutional Review Board
ROC: Receiver operator curve
USD: United States dollar
WHO: World Health Organisation.
